# Chlorhexidine Gluconate Irrigation in the Prevention of Surgical Site Infection Following Cesarean Delivery

**DOI:** 10.7759/cureus.80615

**Published:** 2025-03-15

**Authors:** Garrison Myers, Marissa M Coutinho, Alicia Huckaby, Katie Maloy, Nancy Buderer

**Affiliations:** 1 Department of Obstetrics and Gynecology, Mercy Health St. Vincent Medical Center, Toledo, USA; 2 Department of Statistics, Nancy Buderer Consulting, LLC, Toledo, USA

**Keywords:** antimicrobial irrigation, cesarean delivery, chlorhexidine gluconate, endometritis, irrigation, irrisept, surgical site infection (ssi)

## Abstract

Objective: To determine if the use of 0.05% chlorhexidine gluconate (CHG) irrigation solution during cesarean delivery (CD) can reduce the post-cesarean surgical site infection (SSI) rate when compared to standard practices without CHG irrigation.

Materials and methods: A single-center retrospective observational study was performed at a community hospital in Toledo, Ohio. Control and treatment periods were defined, and patients in the treatment group received irrigation with CHG solution during CD, while the control group did not. An electronic medical record review was conducted to note additional perioperative procedures that affect SSI rates. Patients were evaluated for infection at standard postoperative appointments using the CDC criteria, and the hospital system’s department of infection prevention provided records of all documented SSIs in both groups. Characteristics of the deliveries and SSIs of each group were compared using the chi-square or Fisher’s exact two-tailed tests.

Results: Data were available for 351 deliveries after the implementation of the CHG protocol, and 432 deliveries were used as the control group. No significant difference in rates of infection was found between the two groups (p = 0.68). There were significantly more endometritis infections diagnosed in the treatment group compared to the control group (four (1.1%) versus zero; p = 0.04). Compared to the controls, the treatment group had fewer deliveries with the use of silver dressing, more deliveries with a negative pressure dressing, and more deliveries with a diagnosis of pregestational or gestational diabetes (p < 0.05). There were no other differences between the groups, including the use of abdominal and vaginal prep, BMI > 35, and perioperative antibiotics.

Conclusion: This single center retrospective observational study revealed no difference in post-cesarean SSI rates when performing intra-abdominal and subcutaneous irrigation with CHG solution. A significant increase in endometritis was observed with the use of CHG irrigation; however, further studies are required to determine the benefit or harm of antimicrobial irrigation in CDs.

## Introduction

Surgical site infections (SSIs) account for approximately one-fifth of all healthcare-associated infections (HAIs) [1]. SSIs are infections of the incision, space, or organ that occur within 30 days of surgery [2]. They are a significant cause of morbidity and mortality with consequences including extended hospital stays, financial disparities, discomfort, and increased demands on healthcare resources [1]. The global pooled incidence of SSI is found to be 2.5% [1]. In the United States, the estimated annual incidence ranges from 160,000 to 300,000, with an overall incidence of 2-5% [3,4]. Reducing the incidence of SSIs has become a priority in inpatient care, serving as a metric of quality improvement and patient safety.

Cesarean delivery (CD) is the most common major operating room procedure performed in the United States each year [5]. The incidence of CD has risen dramatically over the last few decades, with a rate of 32.4% of all childbirths in 2023 [6]. As the rate of CD continues to rise, it is also found to be the most significant risk factor of postpartum infection, with infection rates that exceed those of other surgical procedures [7]. The incidence of SSIs in CD is found to be as high as 9% [8].

It has been estimated that approximately half of SSIs are preventable with evidence-based strategies [9]. Commonly used strategies for such prevention in CDs include antibiotic prophylaxis, antimicrobial skin and vaginal cleaning agents, postoperative antibiotic prophylaxis, and negative pressure wound dressings [10-13]. Many novel approaches to SSI prevention are trialed across surgical specialties with an ongoing need to decrease frequency, including the use of antiseptic irrigation.

Chlorhexidine gluconate (CHG) is an antiseptic agent used in a wide spectrum of clinical applications. Chlorhexidine is rapidly absorbed by bacterial cells and causes cytological changes that affect cell permeability, disrupting the osmotic equilibrium and inhibiting growth [14]. CHG has broad activity against gram-positive and gram-negative bacteria, facultative aerobes and anaerobes, and yeast [15]. The only Food and Drug Administration (FDA)-approved CHG-containing irrigation solution is 0.05% CHG in sterile water. This CHG solution has been shown to loosen particulate debris, reduce bacterial load, and inhibit microbial growth [16]. Diluted CHG irrigation is an emerging technique to reduce SSIs, with encouraging outcomes across multiple surgical specialties. In two prospective studies evaluating SSI rates following 0.05% CHG irrigation solution for pilonidal disease and loop ileostomy, patients who received irrigation solution had lower rates of SSIs compared to those who received irrigation with saline alone (p < 0.001) [17,18].

Outcomes following the use of 0.05% CHG irrigation solution in CD remain largely unknown. Thus far, obstetric societies such as the American College of Obstetricians and Gynecologists (ACOG) have not provided recommendations on the use of irrigation of any kind during the standardized CD. This retrospective observational study was commenced to assess if 0.05% CHG in sterile water jet lavage irrigation solution during CD reduced the post-cesarean SSI rate at a single institution.

## Materials and methods

A single-center retrospective observational study was performed at a 591-bed community hospital in Toledo, Ohio. This study was designed to determine if performing surgical irrigation with a 0.05% CHG solution decreased the SSI rate for CD following the implementation of a departmental protocol. The control period was defined as March 2022 through November 2022, and the treatment period was between March 2023 and November 2023. Patients who underwent a CD during the study periods were included. Exclusion criteria included emergency CD and a pre- or intraoperative diagnosis of chorioamnionitis. All operations were performed by one of 10 obstetricians and gynecologists (OBGYN) attending physicians.

The study proposal was submitted to the healthcare system’s research director who determined that the project was studying the systematic implementation of a new department protocol based on evidence-based practice with a US FDA-approved 510k, class II jet lavage medical device (Irrisept®, Irrimax Corporation, Lawrenceville, GA) and did not require an Institutional Review Board (IRB) review.

The treatment period was determined as a period following the initiation of a departmental protocol to perform intraoperative irrigation with 0.05% CHG solution as an initiative to reduce SSIs. The control period was determined as a period prior to the use of antiseptic irrigation and included patients who underwent CD by the same group of physicians the year prior. At the time of the chosen control period, there was no departmental protocol for intraoperative or incisional irrigation and no use of antimicrobial irrigation. The treatment period SSI rates were compared with SSI rates among patients in the control group. The electronic medical record (EMR) was accessed to record study variables, including patient characteristics and procedural factors, as seen in Table 1.

**Table 1:** Characteristics of deliveries after the implementation of the CHG protocol compared to before. Results are shown as frequency count (column percent). Groups are compared using the chi-square or *Fisher’s exact test. X2, df is the chi-square test statistics, degrees of freedom, or Fisher’s exact table probability, cell frequency. The effect size is the Phi coefficient. The p-value is two-tailed. CHG: chlorhexidine gluconate; DM: diabetes mellitus.

	Treatment group, after implementation of CHG protocol	Control group, before protocol	X^2^, df	Effect size	p-value
No. of deliveries	351	432			
Delivery dates	3/22/23 - 12/2/23	3/1/22 - 11/30/22			
0.05% CHG irrigation (Irrisept)	351 (100%)	0 (0%)			
Ancef	339 (96.6%)	412 (95.4%)	0.724, 1	-0.030	0.39
Azithromycin	189 (53.9%)	242 (56.0%)	0.369, 1	0.022	0.54
ChloraPrep abdominal prep	348 (99.2%)	427 (98.8%)	0.259, 3	-0.015	0.74*
Vaginal prep	348 (99.2%)	429 (99.3%)	0.304, 3	0.009	1.0*
BMI > 35	194 (55.3%)	215 (49.8%)	2.350, 1	-0.055	0.13
Silver	156 (44.4%)	245 (56.7%)	11.666, 1	0.122	<0.001
Prevena	177 (50.4%)	171 (39.6%)	9.223, 1	-0.109	0.002
Pregestational, gestational DM	85 (24.2%)	73 (16.9%)	6.439, 1	-0.091	0.01
Any post-op antibiotic	224 (63.8%)	285 (66.0%)	0.395, 1	0.022	0.53
Postoperative antibiotics					
None	127 (36.2%)	147 (34.0%)			
Keflex/Flagyl	173 (49.3%)	187 (43.3%)			
Ancef	42 (12.0%)	69 (16.0%)			
Ancef/Keflex/Flagyl	2 (0.6%)	5 (1.2%)			
Ampicillin/gentamycin +/- clindamycin	2 (0.6%)	2 (0.5%)			
Other	5 (1.4%)	22 (5.1%)			

At the time of CD, all patients in the treatment period received irrigation with 450 mL of 0.05% CHG solution in accordance with the manufacturer’s recommendation for use. Irrigation was performed twice during each procedure: intra-abdominal irrigation following closure of the hysterotomy and incisional irrigation following closure of the fascia. Approximately half (225 mL) of the solution was used intra-abdominally and half after fascia closure. The solution was allowed to sit for 60 seconds prior to removal with suction. Patients in the control period did not receive 0.05% CHG solution, nor was there a standardized protocol for irrigation. Therefore, patients in the control period only received irrigation with normal saline (0.9% sodium chloride) at the discretion of the individual surgeon.

A thorough EMR review was conducted to note perioperative procedures that affect SSI rates, including perioperative antibiotic prophylaxis, antimicrobial skin and vaginal cleansing, wound dressing, BMI, and diagnosis of diabetes. All patients in both the treatment and control groups received antibiotic prophylaxis with weight-based dosing of intravenous cefazolin unless contraindicated secondary to a history of drug allergies. Patients with beta-lactam allergies received 900 milligrams (mg) of clindamycin and weight-based doses of gentamicin. Most patients who underwent a non-elective CD received additional prophylaxis with 500 mg intravenous azithromycin based on evidence from the Cesarean Section Optimal Antibiotic Prophylaxis (C/SOAP) trial [19]. Antibiotic administration time was within one hour prior to incision, compliant with the CDC and ACOG’s recommendations. All patients received the departmental protocol for skin and vaginal preparation. Skin preparation included two applicators containing 2% CHG and 70% isopropyl alcohol solution (ChloraPrep™, Becton, Dickinson and Company, Franklin, NJ). Vaginal preparation was performed using 4% CHG solution (DYNA-HEX 4™, Xttrium Laboratories, Mount Prospect, IL). Patients who underwent emergency CD did not receive standard skin and vaginal preparation; however, all emergency CDs were excluded from the study. The postoperative course was found to have been standardized for most patients in each group. Record review included noting the type of external wound dressing, including antimicrobial silver dressing (Optifoam®, Medline, Northfield, IL) or negative pressure dressing (3M™ Prevena™ Peel and Place Incision Management System, 3M™, Saint Paul, MN). As all OBGYN physicians at the facility routinely administer postoperative antibiotics for patients with a BMI > 35, the investigation of the perioperative course included noting patients who received such prophylaxis.

Patients were evaluated for infection at standard postoperative appointments using the CDC criteria for superficial, deep, and organ space SSIs [2]. The hospital system’s department of infection prevention provided records of all documented SSIs in both the control and treatment periods, allowing a thorough review of the clinical presentation and diagnosis. The diagnosis of each infection was made by treating physicians and confirmed by the department of infection prevention.

All data collected from EMR were entered into a Microsoft Excel spreadsheet (Microsoft Corporation, Redmond, WA). Characteristics of the deliveries and SSIs are presented with frequency counts and percentages and compared between treatment and control groups using the chi-square or Fisher’s exact two-tailed tests. The binomial exact 95% confidence interval (CI) for the infection rate within each group was also calculated. Logistic regression was used to calculate the odds ratio and 95% CI for infection, adjusting for the univariate different characteristics between the treatment and control groups. Data were analyzed with SAS v9.4 (SAS Institute, Cary, NC).

## Results

Data were available for 351 deliveries after the implementation of the CHG protocol (“treatment group”), and 432 deliveries were used as the “control group.” The groups had similar rates of infection (p = 0.68). The treatment group had 15 deliveries with an infection (4.3%) (95% CI: 2.4%, 7.0%). The control group had 16 deliveries with an infection (3.7%) (95% CI: 2.1%, 5.9%). Superficial incisional infections were the most common type in both groups, with 11 (3.1%) in the treatment group and 14 (3.2%) in the control group (Table 2). There were no intra-abdominal abscess infections in the treatment group (0%) and two in the control group (0.5%). When stratifying for these two types of SSIs, there was no significant difference in superficial incisional (p = 0.93) or intra-abdominal abscess (p = 0.50) infections between groups. There were significantly more endometritis infections in the treatment group compared to the control group (four (1.1%) versus zero; p = 0.04).

**Table 2:** Types of infection. Results are shown as frequency count (column percent). Groups are compared using the chi-square or *Fisher’s exact test. X2, df is the chi-square test statistics, degrees of freedom, or Fisher’s exact table probability, cell frequency. The effect size is the Phi coefficient. The p-value is two-tailed. CHG: chlorhexidine gluconate.

	Treatment group, after implementation of CHG protocol	Control group, before protocol	X^2^, df	Effect size	p-value
No. of deliveries	351	432			
Superficial incisional	11 (3.1%)	14 (3.2%)	0.007, 1	0.003	0.93
Endometritis	4 (1.1%)	0 (0%)	0.040, 347	-0.080	0.04*
Intra-abdominal abscess	0 (0%)	2 (0.5%)	0.304, 351	0.046	0.50*

Compared to the controls, the treatment group had fewer deliveries with the use of silver dressing, more deliveries with a negative pressure dressing (Prevena), and more deliveries with a diagnosis of pregestational or gestational diabetes, all with p < 0.05. There were no other differences between the groups, including the use of Ancef, azithromycin, ChloraPrep abdominal prep, vaginal prep, BMI > 35, and postoperative antibiotics (Table 1). Despite the group differences in the use of silver dressing, Prevena, and pregestational/gestational diabetes, the odds of an infection between treatment and control were not significant. The adjusted odds ratio for infection was 1.008 (95% CI: 0.487, 2.087, X2 = 0.0004, df = 1, p = 0.9836).

## Discussion

Considering the impact of SSIs on patients and hospital systems, a continued effort to discover novel approaches to SSI prevention is necessary; however, efficacy, availability, and cost should be highly considered when choosing new protocols for infection prevention. Intraoperative irrigation with 0.05% CHG has been associated with lower SSI rates in surgical practices, including pilonidal disease excision and loop ileostomy [17,18]. Overall, 0.05% CHG irrigation has been studied in multiple surgical specialties with mixed results. In this single-center retrospective observational study, a total of 783 CDs were examined, ultimately finding that intraoperative irrigation with 0.05% CHG solution had no significant impact on the prevention of SSI in CD (p = 0.68).

This single-center study included controlling for standardized practices in the reduction of SSIs, including abdominal and vaginal cleansing and antibiotic prophylaxis in both the pre and postoperative state. As CDs include a risk of infection from both skin and vaginal infectious organisms, antimicrobial skin and vaginal cleansing agents are used as prevention. The CDC recommends preoperative skin cleansing with an alcohol-based solution [11]. Antiseptic cleansing of the vagina preoperatively has also been adopted into most practices and found to reduce endometritis and postoperative fever in patients laboring or with ruptured membranes; however, it has not been found to decrease wound infections [12]. In this study, emergency CDs were excluded due to the increased risk of infection, partially due to the lack of skin and vaginal cleansing. As a result, most patients in both the treatment and control group had documentation of receiving the recommended antimicrobial skin (99.2% and 98.8%, respectively) and vaginal cleansing (99.2% and 99.3%) prior to delivery.

Preoperative weight-based antibiotic prophylaxis administered within 60 minutes of the start of the surgery is recommended for all CDs [20]. A Cochrane review has found significant reductions in both wound complications and endometritis with the use of antibiotic prophylaxis in emergent and nonemergent CDs [10]. Novel approaches to SSI reduction in nonelective CDs include administration of 500 mg of intravenous azithromycin in addition to standard antibiotic prophylaxis, which has been shown to significantly reduce overall infection rates in a randomized controlled trial [21]. In this study, all patients in the treatment and control group received preoperative antibiotic prophylaxis, and approximately half of the patients in the treatment and control groups received recommended azithromycin for nonelective CD (53.9% and 56.0%, respectively).

The use of postoperative oral antibiotic prophylaxis following CD in obese patients is rising. A recent clinical trial revealed a significant decrease (6.4% versus 15.4%) in SSI following a 48-hour postoperative course of oral cephalexin and metronidazole after also receiving standard preoperative prophylactic antibiotics. The study was limited by patients receiving additional preoperative antimicrobial prophylaxis with azithromycin; therefore, the overall benefit of postoperative oral antibiotics remains unclear [13]. In this study, approximately two-thirds of the treatment and control groups received postoperative antibiotics (63.8% and 66.0%, respectively).

Although wound irrigation is a popular procedure in surgical practice, the lack of overall procedure standardization leads to heterogeneity in available evidence [22]. Intraoperative wound irrigation has been studied in a wide variety of surgeries, including breast, pilonidal, and bowel surgery, and has been found to decrease SSI. The incidence of intra and postoperative morbidity has also been explored. Recent literature suggests that irrigation during CD is associated with increased intraoperative nausea and increased use of postoperative antiemetics, with no meaningful decrease in postoperative morbidity, including SSI [23]. Increased rates of operative discomfort and nausea are thought to be due to peritoneal irritation. The use of intraperitoneal irrigation during CDs continues to be controversial, given the mixed data on risk versus benefit and possible morbidity [23]. Obstetric societies, including ACOG, lack recommendations for irrigation use of any kind in the standardized CD. In this study, although all deliveries in the treatment group received intra-abdominal and incisional irrigation with 0.05% CHG solution, further irrigation with normal saline was not standard and at the discretion of the individual surgeon. In addition, there was no standardized use of normal saline irrigation during the time of the control group. Yet, the study found no decrease in SSI after irrigation with CHG solution when compared to the control group. Given the unique nature of CD, with patients being conscious for the duration of the operation, it is important to consider the irritating effects of irrigation, causing an increase in intraoperative morbidity and a negative birthing experience for patients. Therefore, when considering the utilization of any irrigation, including antimicrobial irrigation, attention to previously published data that suggest an increased morbidity is necessary.

Different wound dressings have been studied as additional techniques to reduce SSI. Our study observed wounds dressed with either silver-impregnated dressings or a negative pressure wound vacuum. Overall, data are inconsistent for silver dressings and the reduction of SSI. Some studies have shown silver-impregnated dressings to reduce SSI after CD, but not to a statistically significant outcome, whereas others found no significant difference in SSI with silver dressings [24,25]. Negative pressure wound therapy (NPWT) is used for wound healing following an infection but is now being studied as a prophylactic measure to prevent SSIs. NPWT is used to remove excess interstitial fluid, increase tissue vascularity, and decrease bacterial colonization. A large, multi-center, randomized control trial studied whether obese patients (defined as BMI > 30) who received a NPWT versus standard dressing (tape and gauze) after CD would reduce SSI rates. This study found no significant difference in SSI rates and was ultimately discontinued due to an observed increased risk of adverse skin reactions [26]. In the present study, there was no institutional policy for dressing type; however, most physicians used a BMI > 35 as a cutoff for using a Prevena wound vacuum. Overall, there was a significant difference in dressing type between the two groups, with the treatment group receiving more Prevena wound vacuum dressings (50.4%, p < 0.002) and the control group receiving more silver dressings (56.7%, p < 0.001) (Table 1). Despite the increased use of NPWT and 0.05% CHG solution in the treatment group, there was no difference in SSIs compared to the control (OR: 1.008, 95% CI: 0.487-2.087). Further studies are warranted to investigate how dressing type can prevent SSI with or without the use of antimicrobial irrigation.

The risk of SSI in association with diabetes and obesity has been widely studied. Recent literature reveals an association between obesity and SSI, specifically in post-cesarean follow-up, while diabetes remains an inconclusive risk factor for SSI [27,28]. One large, multi-center, prospective cohort study found an increased risk of post-cesarean SSIs in obese populations, defined as a BMI >30; however, the risk of SSI and diabetes was not significant when controlled for the presence of obesity [27]. In this study, there were significantly more patients with pregestational or gestational diabetes in the treatment group (p < 0.01); however, no statistical difference in BMI > 35. Approximately 65% (10 of 15) of the SSIs found in the treatment group were in patients with diabetes. In comparison, 19% (three of 16) of SSIs in the control group were in patients with diabetes. Despite the increase in diabetes in the treatment group, there was no difference in SSIs compared to the control (OR: 1.008, 95% CI: 0.487-2.087). The question remains whether an increased rate of diabetes in the treatment group could increase the risk of SSI, therefore altering this study's ability to determine the 0.05% CHG solution’s effect on SSI prevention; however, current data remain inconclusive to show diabetes alone as a risk factor for SSI after CD. Overall, it is difficult to identify the efficacy of 0.05% CHG solution in the obese and diabetic populations given their independent risk factors for SSI.

Endometritis is an infection of the uterus and complicates the postoperative period of CDs 6-27% of the time [29]. Although prevention of endometritis does not differ from other SSI preventative methods before or after CD, preoperative vaginal cleansing has been found to play a significant role in decreasing the risk of post-cesarean endometritis. A recent Cochran review revealed a more than 50% decrease in endometritis with practicing preoperative vaginal cleansing [30]. As discussed above, there was no significant difference in preoperative vaginal cleansing between groups (99.2% vs. 99.3%); however, the rate of endometritis differed. In this study, there were no reported cases of post-cesarean endometritis in the control group; however, the treatment group experienced four cases of diagnosed endometritis (1.1% versus 0%; p = 0.04). Of note, three of the cases of endometritis were diagnosed clinically, with only one case being positive for gram-positive cocci on culture. While previous studies with CHG vaginal prep have found to decrease inflammation and endometritis [12], there is no current data to determine if 0.5% CHG intra-abdominal irrigation has an effect on inflammation. One hypothesis for the observed increase in endometritis relates to previous studies citing peritoneal irritation caused by intra-abdominal irrigation [23]. It is possible that irrigation with Irrisept caused peritoneal irritation and, in the postoperative state, manifested as uterine or fundal tenderness. As a result, a higher number of clinical diagnoses of endometritis without culture-proven results were seen in the treatment group. It seems unlikely for antimicrobial irrigation to be an independent cause of endometritis as it was applied after hysterotomy closure and should not interface with the vaginal flora or endometrium. Future studies are needed to determine the risk of endometritis following intra-abdominal irrigation with 0.05% CHG solution after the closure of the hysterotomy and prior to the closure of fascia.

The cost of perioperative procedures used to prevent SSIs varies; however, such measures typically have fewer financial implications than unplanned wound complications and hospital readmission [3]. The 0.05% CHG irrigation solution used in this study (Irrisept) cost US$ 65 per patient for one-time use, which can be compared to other SSI preventative measures at the institution. Irrigation with normal saline solution costs US$ 2.13 per patient per CD, silver dressing costs US$ 24.44 per one-time use, and negative pressure dressing costs US$ 499.99 per one-time use. Although the negative pressure dressing is the most expensive, the CHG irrigation solution increases cost compared to other preventative measures. Operating room (OR) time can also be considered when discussing cost and resource burden in the prevention of SSIs. Although OR time was not measured in this study, it can be presumed that at least two minutes of increased OR time is experienced when using 0.05% CHG irrigation solution twice per procedure if following the manufacturer's recommendations of allowing the solution to sit for 60 seconds [16]. Although the 0.05% CHG irrigation solution used in this study (Irrisept) does not pose a significant consequence of financial or resource-burden, the efficacy of the solution should be considered and further studied to determine if the benefit outweighs the cost.

Limitations of this study include general applicability due to a small sample size and a retrospective review of a single center. Therefore, the authors acknowledge that results may not be generalizable to other patient populations or institutions. A power calculation was not conducted before the study. The study was limited to the deliveries at one institution during pre-specified periods. There was only a 0.6% difference in the infection rates between the treatment and control groups. This is a clinically insignificant difference. Post hoc power analysis shows that a sample size of 16,744 deliveries per group would be needed to detect this size difference with 80% power and 5% type I error. With the available sample sizes, the study could detect a difference of 4.8% or more between groups (e.g., 8.5% vs. 3.7%). Irrigation techniques and inter-surgeon differences were not standardized in the control group and the authors recognize this occurred due to the retrospective nature of the study and can potentially confound results of SSI rates.

## Conclusions

This single-center retrospective observational study revealed no difference in post-cesarean SSI rates when performing intra-abdominal and subcutaneous irrigation with 0.05% CHG solution. Although it is unlikely for antimicrobial irrigation to be an independent cause of endometritis, this study observed a significant increase in endometritis with the use of CHG irrigation. Further studies are required to determine any benefit or risk of CHG irrigation in preventing post-cesarean SSIs.
